# Colonization with non-mycorrhizal culturable endophytic fungi enhances orchid growth and indole acetic acid production

**DOI:** 10.1186/s12866-022-02507-z

**Published:** 2022-04-13

**Authors:** Sujit Shah, Biva Shah, Rohit Sharma, Bhagwan Rekadwad, Yogesh S. Shouche, Jyotsna Sharma, Bijaya Pant

**Affiliations:** 1 Central Department of Botany, Tribhuvan University, Kathmandu, 44613 Nepal; 2 Daffodil Agro Biological Research Center, Lalitpur, 44700 Nepal; 3 National Centre for Microbial Resource, National Centre for Cell Science, NCCS Complex, Savitribai Phule Pune University Campus, Ganeshkhind, Pune, 411007 India; 4 School of Sciences, SAGE University, Katara Hills, Bhopal, MP India; 5 Division of Microbiology and Biotechnology, Yenepoya Research Centre, Yenepoya (Deemed to be University), Mangalore, Karnataka 575018 India; 6 Department of Plant and Soil Science, Texas Tech University, Box 42122, Lubbock, TX 79409 USA

**Keywords:** Endophytes, Phytohormones, Auxin, Bioactive compounds, Root length, Shoots length, High performance liquid chromatography, Scanning electron microscopy

## Abstract

**Background:**

Symbiotic associations of endophytic fungi have been proved by possessing an ability to produce hormones and metabolites for their host plant. Members of the Orchidaceae are obligate mycorrhizal species but a non-mycorrhizal association needs more investigation for their ability to promote plant growth and produce plant growth hormones. In the present study, endophytic fungi were isolated from the roots of *Dendrobium longicornu* Lindl., to investigate the root colonizing activity and role in plant growth and development.

**Results:**

Among 23 fungal isolates were identified both by morphological and molecular technique as *Penicillium* sp., *Fusarium* sp., *Coniochaeta* sp., *Alternaria* sp., and *Cladosporium* sp. The dominate species were *Coniochaeta* sp. and *Cladosporium* sp. The dominant species as per the isolation was *Coniochaeta* sp. These fungal strains were screened for growth-promoting activity of *Cymbidium aloifolium* (plantlet) consider as cross genus interaction and *Dendrobium longicornu* (protocorms) as a host plant in in-vitro condition. Importantly, *Cladosporium* sp., and *Coniochaeta* sp. showed successful colonization and peloton formation with roots of *C. aloifolium*. Moreover, it also enhanced acclimatization of plantlets. Fungal elicitors from nine fungal isolates enhanced the growth of the in vitro grown protocorms of *D. longicornu*. Key bioactive compounds detected in the fungal colonized plant extract were 2H-pyran-2-one, Cyclopropanecarboxylic acid, Oleic Acid and d-Mannitol, which may have a potential role in plant-microbe interaction. All fungal endophytes were able to synthesize the indole acetic acid (IAA) in presence of tryptophan. Moreover, fungal extract DLCCR7 treated with DL-tryptophan yielded a greater IAA concentration of 43 μg per ml than the other extracts. The *iaa*M gene involved in IAA synthesis pathway was amplified using *iaaM* gene primers successfully from *Alternaria* sp., *Cladosporium* sp., and *Coniochaeta* sp.

**Conclusions:**

Hence, this study confirms the production of IAA by endophytes and demonstrated their host as well as cross-genus plant growth-promoting potential by producing metabolites required for the growth of the plant.

**Supplementary Information:**

The online version contains supplementary material available at 10.1186/s12866-022-02507-z.

## Background

Orchid mycorrhizal endophytes are the key microbes for the conservation of orchid species [1, 2]. Root-associated endophytes are known to promote plant growth through nutrient supplementation. The orchids, an obligate mycorrhizal group of plants belonging to one of the largest angiosperm families, require mycorrhizal association of its germination and propagation. Orchids and microbes share a chemical ecology, and the evidence shows that fungi-synthesizing auxin and phenolic compounds play an important role in plant-microbe interaction [3, 4]. Diverse and novel fungal species have been explored from the orchids [4–6]. *Cymbidium aloifolium* (L.) Sw. (aloe-leafed) and *Dendrobium longicornu* Lindl., (long-horned) are the known ornamental and medicinal orchid; epiphytic on moss-covered humid trees of the coniferous forest; distributed in the hills of Nepal, Southern part of China, and Northern part of India [7].

The mass propagation of orchid species in vitro as well as in-vivo condition is challenging. Most of the orchids grown in tissue culture media like Murashige and Skoog medium with different nutrient supplements and conditions still have slow seed germination and growth. Modifications in MS media, cofactors and hormonal composition have been used to improve orchid growth under in vitro conditions [7–12]. However, the growth still remains slow in consideration with a height of the plantlets, root and shoot number as well as length. Similarly, plantlets suffer from intense biotic and abiotic stress resulting in high mortality during the acclimatization [5]. Microbes association can play a major role during acclimatization. The importance of mycorrhizal fungi in plant growth has been extensively established. Despite the fact that non-mycorrhizal fungi can produce antioxidant compounds and phytohormones, additional research into their impact on plant growth is needed. Most of these non-mycorrhizal endophytes belong to phylum Ascomycota and reside in leaves, steam, flower and roots [13–20]. The present study hypothesizes that non-mycorrhizal endophytes may have a significant role in root colonization, bioactive compound and IAA production and promote plant growth. In this regard, the present study was undertaken to isolate and identify endophytic fungi residing in the leaves and roots of the three wild plants of *D. longicornu* growing on *Quercus semecarpifolia* Sm, *Pyrus pashia* Buch. & Ham and *Schima wallichii* Choisy and investigated their ability to colonize the root of orchid, ability to produce indole acetic acid, bioactive compounds for the growth and development of orchid plantlets in our experimental settings.

## Results and discussion

### Molecular identification of endophytic fungi

Endophytic IAA producing fungi isolated from the *D. longicornu* were identified by ITS sequencing method. A total of 23 isolates were isolated of which DLCR1, DLCR2 and DLCR3 were identified as *Penicillium* sp., PDLAR1, PDLAR2 and PDLAR3 were identified as *Fusarium* sp., DLCCR3 and DLMR3 as *Coniochaeta* sp., CDLAR1 and DLCL2 as *Alternaria* sp., and DLCCR7 as *Cladosporium* sp. The strain DLCCR7 isolated from the root of *D. longicornu* showed 92% identity with *C. africana*. The DLCCR7 has been characterized as *Coniochaeta dendrobiicola* as isolated from the root of the *D. longicornu* by our team [21]. The DLCCR7 fungal isolate was deposited in National Centre for Microbial Resource with an accession number MCC1811 and MycoBank accession number MB830652. The consensus ITS sequence of each identified fungus has been deposited in the NCBI database (Table 1). These endophytes are reported to be as non-mycorrhizal endophytes belonging to phylum Ascomycota. These endophytes are reported to be beneficial endophytes for the plant growth [13–20]. However, role *Coniochaeta* sp. as beneficial microbes have not been reported.

**Table 1:** The fungal isolates and their codes along with their taxonomy and gene accession number

Fungal Morphotype code	Fungal taxonomy	Number of isolates	Percentage of identity	Gene accession number
CDLAR1	*Alternaria* sp.	1	100%	MN256650
DLCCR3	*Cladosporium* sp.	4	99%	MN256649
DLCCR7	*Coniochaeta* sp.	4	92%	MK225602
DLCL2	*Alternaria* sp.	2	100%	MN256652
DLCR1	*Penicillium* sp.	1	100%	MN256653
DLCR2	*Penicillium* sp.	2	100%	MN256654
DLCR3	*Penicillium* sp.	1	100%	MN256648
DLMR3	*Coniochaeta* sp.	3	100%	MN256651
PDLAR1	*Fusarium* sp.	2	98%	MN256645
PDLAR2	*Fusarium* sp.	1	100%	MN256647
PDLAR3	*Fusarium* sp.	2	100%	MN256646

*DLCR* represent ‘DL’ *Dendrobium longicornu*, ‘C’ Czapek Dox Agar, ‘R’ Root. *DLCCR* represent ‘DL’ *Dendrobium longicornu*, ‘C’ Chilaune, ‘C’ Czapek Dox Agar, ‘R’ Root. ‘L_2_’ *DLCL*_*2*_ represent leaf. *DLMR* represent ‘DL’ *Dendrobium longicornu*, *‘*M’ MMN media, ‘R’ Root. *CDLAR* represent ‘C’ Czapek Dox Agar, ‘DL’ *Dendrobium longicornu*, ‘A’ Maile, ‘R’ Root. *PDLAR* represent ‘P’ PDA media, ‘DL’ *Dendrobium longicornu,* ‘A’ Maile, ‘R’ Root

### Indole acetic acid (IAA)

The *iaa*M gene was amplified from endophytic fungus DNA and gave a 400-bp consensus. Manual quality control was performed on tryptophan-2-monooxygenase (*iaa*M) gene sequences, and consensus was deposited in the NCBI repository under accession numbers MK281634 (DLCCR3), MK281635 (DLCCR7), MK281636 (DLMR3), and MK281639 (CDLAR1). In this case, the *iaa*M gene was amplified using a *Fusarium*-specific *iaa*M primer, DLCCR3, DLCCR7, and DLMR3 [22]. The findings further demonstrate that the *Fusarium*-specific *iaa*M primer is successful in amplifying *iaa*M sequences in fungi of any species.

### Biochemical assay

#### Quantification of indole acetic acid (IAA)

**Fig. 1:**
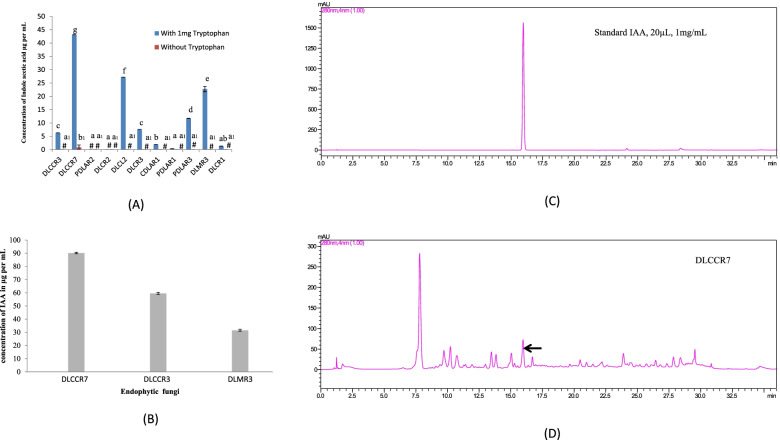
**A** Auxin synthesis by endophytes with and without tryptophan induction. Out of a total of 11 fungal endophytic isolates, eight produced IAA in the presence of tryptophan induction broth. **B** Out of a total of 11 fungal endophytic isolates, the three species were selected and their corresponding isolates were subjected to repeated IAA quantification. Values with different letters are significantly different at *p* ≤ 0.05 (Tukey test), **C** chromatogram of standard IAA as control and **D** Arrow indicate IAA detection peak in DLCCR7 broth

Indole acetic acid estimation revealed variable concentrations of indole acetic acid (IAA) in the culture broth prepared from endophytic fungi. IAA concentration was detected in broths supplemented with tryptophan for all endophytes except PDLAR2 and DLCR2 whereas the presence of IAA was negligible without tryptophan. The fungal extract DLCCR7 treated with DL-tryptophan yielded a greater IAA concentration of 43 μg per ml than the other extracts as shown in Fig. 1A.

#### Quantification of IAA and metabolomics

Out of a total of 23 fungal isolates, one was selected from each fungal taxon making a total of 11 species subjected to quantification of IAA based on the identification and isolation tissue source. These three isolates (DLCCR7, DLCCR3 and DLMR3) were subjected to the 2nd round of IAA quantification as shown in Fig. 1B as these isolates were able to produce IAA and colonized the plant as shown in the plant growth assay section. The standard IAA chromatograph was previously published [23]. The HPLC analysis of IAA revealed that DLCCR7 had the highest concentration, followed by DLCCR3 and DLMR3. DLCCR7 extract has the highest content of IAA at 90 μg per ml, followed by DLCCR3 and DLMR3 (Fig. 1B). Figure 1C and D show the chromatograms of the standard IAA solution and the extract DLCCR7, respectively. According to reports, indole compounds operate as a communicator or signaling molecules in plant-fungi interactions [3, 24].

### Detection of bioactive compounds

The chromatograph of the bioactive compound identified from an uncolonized plant was previously published [25]. GC-MS analysis of extract prepared from uncolonized plantlets, fungal extract (Fig. S3 and Table S1, Figs. S7-S9) and colonized plantlets are depicted in Table 2. The main metabolites detected in plants colonized by fungal isolates were n-Hexadecanoic acid, Cyclopropane carboxylic acid, Eicosadienoic acid, d-Mannitol, Oleic Acid, 2H-Pyran-2-one, Triazole. As these compounds were present in the plant interacted with fungi. The compounds could be involved in plant-fungal communication, plant growth regulation, and antimicrobial activity. The plant colonized by DLCCR7 and DLCCR3 contained n-Hexadecanoic acid reported as an antimicrobial compound [26]. The cyclopropane carboxylic acid was found in Plant colonized by DLMR3, DLCCR7, and DLCCR3 extract and was reported to be a precursor of ethylene production [27]. Similarly, a compound having biotic stress tolerant activity such as Oleic acid [28] was detected in plants colonized by DLCCR3 and DLCCR7. Similarly, 2H-pyran-2-one have been reported for root growth and development and this compound was present in plants colonized by Plant-DLCCR7, Plant- DLCCR3 [29]. Whereas Triazole (biotic stress controller) [30], eicosadienoic acid (biotic stress controller) [31], d-Mannitol (abiotic and biotic stress controller) [32] were only found in plants colonized by DLMR3.

**Table 2:** A comparison of metabolite identified in the methanolic extract of endophytic fungi colonized and uncolonized *Cymbidium aloifolium* plantlet

Name of Compound	Fungal Extract	Uncolonized Plant	Colonized Plant	Function
n-Hexadecanoic acid	DLCCR7 DLCCR3	P	Plant-DLMR3, Plant-DLCCR7	Antimicrobial activity [26]
Cyclopropanecarboxylic acid	DLCCR7, DLCCR3	NP	Plant-DLMR3, Plant-DLCCR7, Plant- DLCCR3	Plant ethylene biosynthesis [27]
Oleic Acid	DLCCR7, DLCCR3	NP	Plant-DLCCR3, Plant-DLCCR7	Biotic stress tolerance [28]
2H-Pyran-2-one,	DLMR3	NP	Plant-DLCCR7, Plant- DLCCR3	Plant growth and Root development [29]
1,2,4-Triazole	DLMR3	NP	Plant-DLMR3	Biotic stress tolerant [30]
11,14-Eicosadienoic acid, methyl ester	NP	NP	Plant-DLMR3	Biotic stress tolerance [31]
d-Mannitol	NP	P	Plant-DLMR3	Abiotic and Biotic stress tolerance [32]
Imidazole, 2-amino-5-[(2-carboxy)vinyl	NP	NP	Plant-DLCCR7	Plant growth regulator [33]
2,3,4,6-Tetra-O-acetyl-D-glucopyranose	DLCCR7	NP	NP	Plant growth and Root development [34]
2-Furancarboxaldehyde, 5-(hydroxymethyl)-	DLCCR7	NP	NP	Antimicrobial activity [35]
Thiazolidin-4-one, 5-ethyl-2-imino-	DLMR3	NP	NP	Antimicrobial activity [36]
6-Acetyl-.beta.-d-mannose	DLMR3	NP	NP	Cell wall development [37, 38]

*NP* Not present, *P* Present

### Plant growth assay with fungal elicitors

**Fig. 2:**
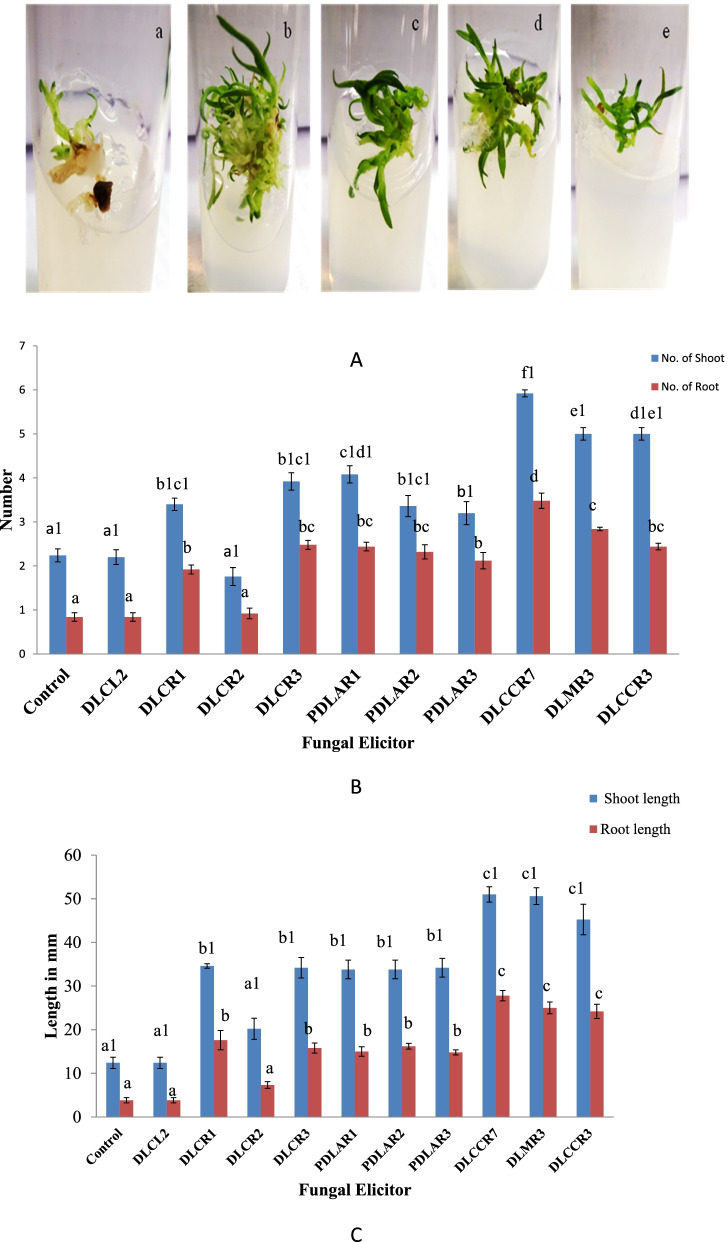
**A** Phenotype of *Cymbidium aloifoilium* grown (a) on Murashige and Skoog (MS) media (control), or in MS media supplemented with fungal elicitors: (b) DLCCR7, (c) DLMR3, (d) DLCCR3 and (e) DLCR2. **B** Growth pattern of the DLCCR7, DLCCR3 DLMR3 colonized plantlet is higher in terms of mean of root and shoot number, **C** as well as mean of root and shoot length. Bar represents mean ± SE (*n* = 15). The Values with different letters are significantly at the level of *p* ≤ 0.05

Endophytic fungi differ from other types of fungi because they have active pathways and expressed genes. The protocorms of *D. longicornu* benefited from a plant growth experiment with a fungal elicitor. Except for DLCL2 and DLCR2, almost every fungal elicitor has boosted protocorm (*D. longicornu*) growth (Fig. 2). The DLCCR3, DLCCR7, and DLMR3 protocorms grew faster in terms of root and shoot number, as well as average length (Fig. 2B & C). The fungal elicitor DLCCR7’s plant growth-promoting activity was considerably higher at *p* > 0.05. In protocorms treated with DLCCR7 elicitor, the average number of shoots and roots was 5.9 and 3.48, respectively. Similarly, the length of the shoot and root in DLCCR7-treated protocorms was 51 mm and 25 mm, respectively. Similarly, in protocorms treated with DLMR3 elicitor, the average number of shoots and roots was 5 and 2.84, respectively. Similarly, the lengths of the shoot and root in DLMR3-treated protocorms were 50 mm and 27.8 mm, respectively. In the protocorms treated with DLCCR3 elicitor, the average number of shoots and roots was 5 and 2.44, respectively. Similarly, the length of the shoot and root in DLCCR3-treated protocorms was 51 mm and 25 mm, respectively. Protocorms treated with elicitors of isolated DLCL2 and DLCR1 grew poorly.

There exist borderline differences between mutualistic and pathogenic fungi. Depending on the genetic makeup of the fungus, environmental factors, and nutrient supply, the mutualism can be interchangeable [24, 39–41]. *Fusarium* sp. has been shown to colonize orchid roots, according to recent research. Similarly, the potential of *Fusarium* sp. to colonize roots when co-cultured with *Bletilla striata* and *Dendrobium candidum* has been observed [42]. The potential of *F. oxysporum* KB-3 to create orchid pelotons within the cortical cells was discovered by microscopic examination of the root section. *Fusarium* sp. has been reported to have considerable plant growth-promoting effects and could be a possibility for bioactive chemical synthesis [43]. Similar evidence that shows *Fusarium* being beneficial endophyte has been demonstrated the interaction of *Fusarium* sp. with maize [44]. They discovered that *Fusarium* plays a function in cytokinin and IAA production. In various orchid species, *Fusarium* endophytes have been shown to promote plantlet growth and seed germination [45–47]. The role of *Penicillium* sp., in plant growth, production of IAA and organic matter as well as phosphate solubilizing activity has been well demonstrated in non-photosynthetic orchid, *Arachnitis uniflora* Phil. [48]. Similarly, *Penicillium* sp. and *Cladosporium* sp., isolated from the *Stanhopea trigrina* have proven to be gibberellin producers [49]. However, there are limited reports regarding the plant growth promoting activity of *Cladosporium* sp. with orchid species.

### Co-culture assay and confirmation of root colonization

**Fig. 3:**
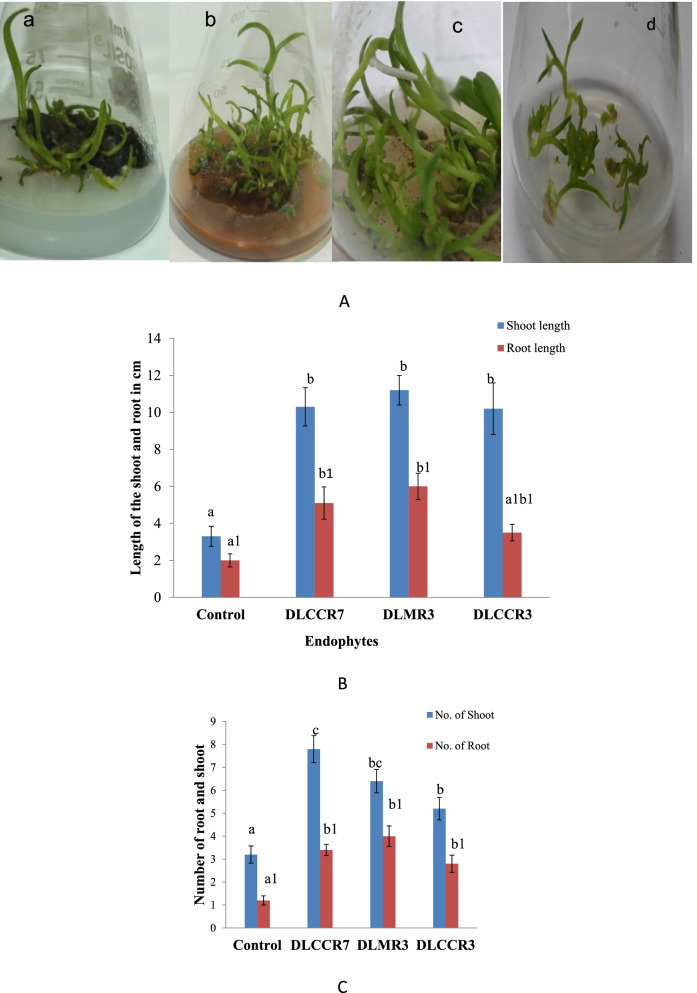
**A** Phenotype of *Cymbidium aloifoilium* grown (a) on Murashige and Skoog (MS) media with DLCCR3, on (b) with DLCCR7, on (c) with DLMR3, (d) MS media (control). **B** Significant increase in plant growth of the plantlets colonized by DLCCR7, DLCCR3 and DLMR3 versus uncolonised plantlets in terms of mean of root and shoot number, **C** as well as mean of root and shoot length. Bar represents mean ± SE (*n* = 15). The Values with different letters are significantly different at the level of *p* ≤ 0.05

**Fig. 4:**
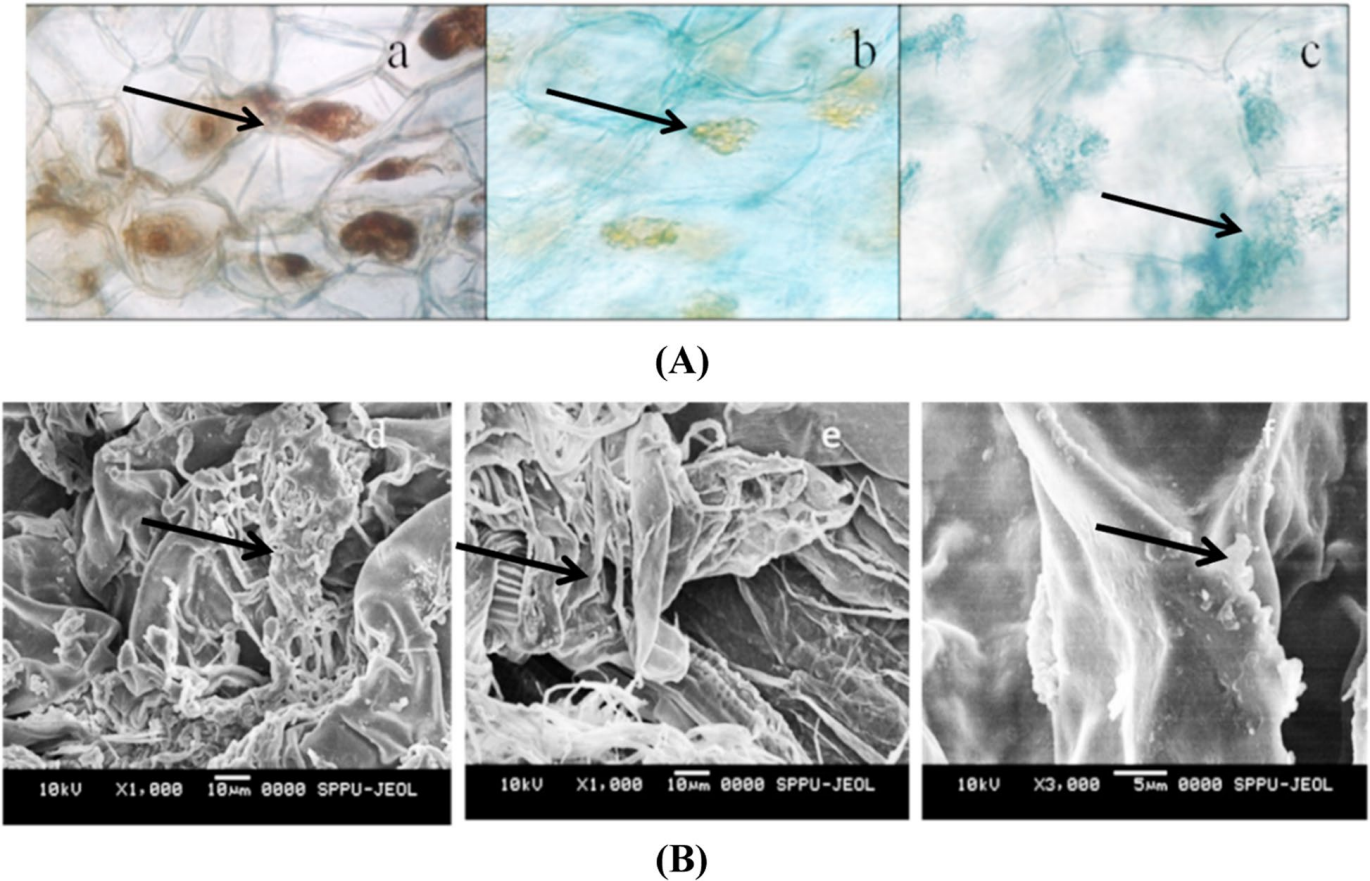
**A** Bright Field Microscopy: The root section showing formation of pelotons (black arrows) in root cortical cells of *Cymbidium aloifoilium* inoculated with (a) DLCCR7, (b) DLCCR3, (c) DLMR3. **B** Scanning Electron Microscopy: The root colonized by fungus; (d) showing the presence of DLCCR7 fungal hyphae in colonized root (black arrows); (e) showing the presence of DLMR3 fungal hyphae in colonized root (black arrows) and (f) showing the presence of DLCCR3 fungal hyphae in colonized root (black arrows)

Irrespective of IAA producing ability, co-culture assay (in vitro) was between *C. aliofolium* plantlets and endophytes isolated during study. Endophytic fungi viz. DLCCR3, DLCCR7 and DLMR3 showed the root colonization and enhanced growth of inoculated plantlets. This experiment was done in five replicates to reproduce results and confirm the outcome of the study (Fig. 3A). Growth of each plant was monitored and compared with control. Morphological changes such as length and number of each shoot and root indicate that significant growth was observed after 60 days. Though there was not much variation in length of shoot among plants inoculated with DLMR3, DLCCR7 and DLCCR3 having average shoot length value 11.2 cm, 10.3 and 10.2 respectively but significantly higher than that of control. The root length was greater in plantlets inoculated with DLMR3 and DLCCR7, with average values of 6 cm and 5.1 cm, respectively, compared to 3.5 cm and 2 cm in plantlets inoculated with DLCCR3 and control, respectively (Fig. 3B). The average shoot number was 7.8, highest in the plantlet inoculated with DLCCR7, whereas almost equal root number was recorded in plantlets inoculated with DLMR3, DLCCR7 and DLCCR3 having average root numbers 4, 3.4 and 2.8 respectively (Fig. 3C) However, sum of root number was greater in inoculated plants than control. Histo-chemical analysis of the root section of the plantlet inoculated with DLCCR3, DLCCR7 and DLMR3 was demonstrated. The roots were stained with 0.05% trypan blue to visualize fungal pelotons under bright field microscope (Fig. 4A). SEM of root section shows fungal pelotons in roots inoculated with DLCCR3, DLCCR7 and DLMR3, which confirms presence of symbiotic association (Fig. 4B).

### Acclimatization

**Fig. 5:**
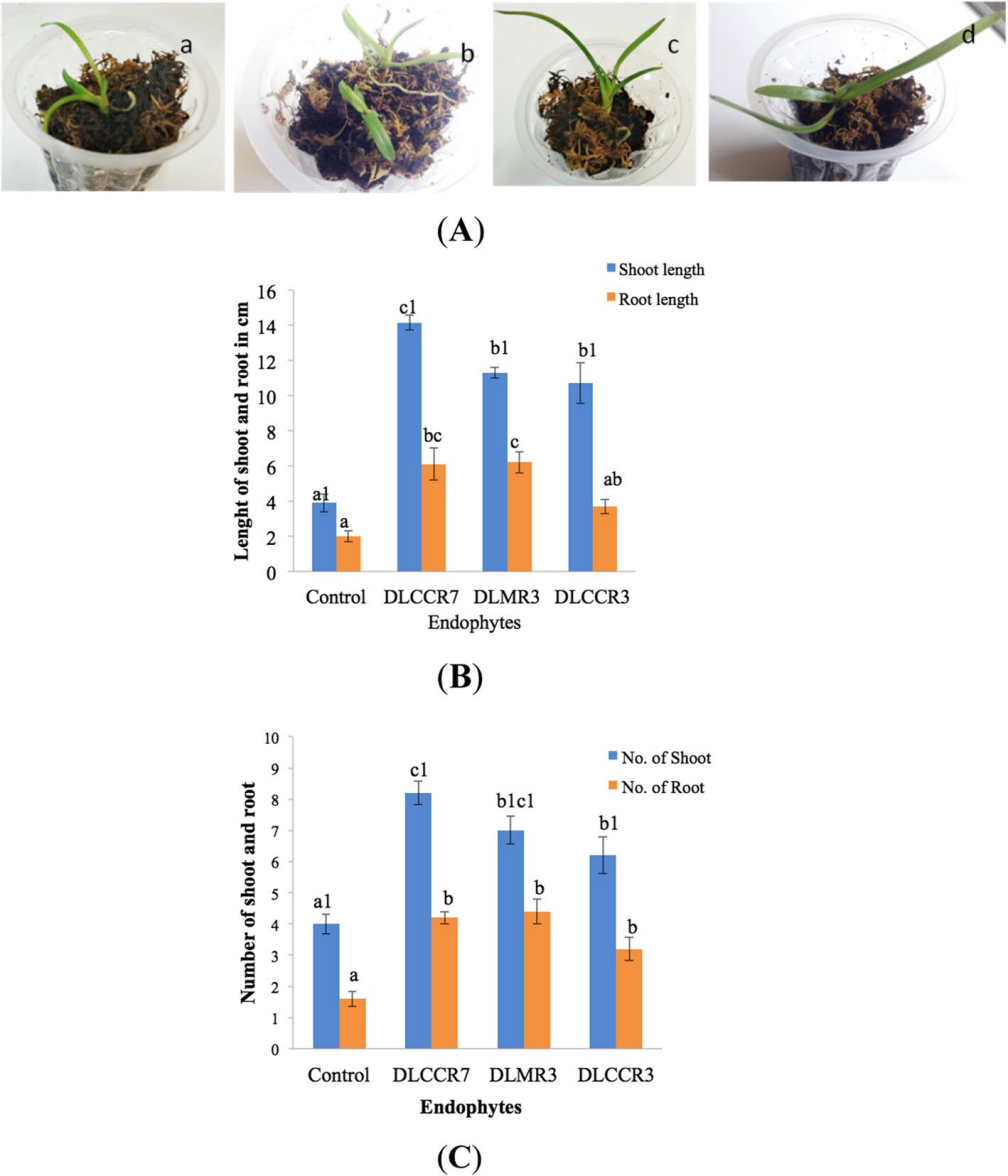
**A** Acclimatization and hardening of the fungus-colonized plants and uncolonized plants for 2 months using coocopeat and moss in ratios 2:1. (a) Control, (b) plant colonized with DLMR3, (c) Plant colonized with DLCCR3 and (d) Plant colonized with DLCCR7. **B** Morphological changes in growth pattern of the DLCCR7, DLCCR3 DLMR3 colonized plantlet versus uncolonised plantlet in terms of mean of root and shoot length as well as **C** mean of root and shoot number. Bar represents mean ± SE (*n* = 10). The Values with different letters are significantly different at the level of *p* ≤ 0.05

Those plantlets possess confirmed presence of symbiotic association between endophytic fungi and experimental plants in former in vitro experiment were transferred to field for in vivo study or hardening. In comparison to the treated plantlets, the control plantlets showed stress and poor development. During the acclimation stage, all of the plants survived. Use of substrate i.e., coco peat and moss (2:1 ratio) shows that the plantlet colonized by DLCCR7 has attained higher growth as compared to the control after 60 days. There was also significant growth in plantlets colonized by DLMR3 and DLCCR3 compared to control (Fig. 5). The co-culture assays put forward new finding showing that DLCCR7 *Coniochaeta dendrobiicola* (isolate DLCCR7), *Coniochaeta* sp. (isolate DLMR3), Cladosporium sp. (isolate DLCCR3) were able to colonize the roots of the in vitro grown *C. aloifolium* plantlets. Moreover, the plants colonized by these fungi adapted well with adverse environmental stress compared to the uncolonised plantlet. The *Coniaochaeta* sp. has been reported to show plant growth promoting activities as well as antifungal and metabolites production [50]. The present study has clearly shown the evidence of root colonizing activities of the isolates with *C. aloifolium* and the fungal extract able to promote the protocorms growth. However, further investigation has to be done to explore about DLCCR7 was identified as novel species *C. dendrobiicola* [21].

## Conclusion

The present study shares the new information in the area of orchid microbes interaction. Endophytes isolated from the roots and leaves considered non-mycorrhizal endophytes and belonging to the phylum Ascomycota. When given as a tonic in-vitro condition, the endophytes isolated from the root were able to colonize the plant roots and promote growth. The isolates from the leaves, on the other hand, were ineffectual for plant growth. The aforementioned experiment concludes that root endophytes differ from leaf endophytes in root colonization and plant growth-promoting activities. The study successfully demonstrated the root colonization ability of fungal isolates considered as non-mycorrhizal endophytes. The study emphasized the plant growth-promoting action of *Coniaochaeta* sp., *Coniaochaeta dendrobiicola*, and *Cladosporium* sp. for orchid species for the first time. *C. dendrobiicola* being the novel species, potential root endophytes. The current investigation demonstrated the accuracy of the *iaa*M primer for *iaa*M gene amplification across fungal species.

## Material and methods

### Collection of samples

Individual of epiphytic *D. longicornu* one on each tree, *Quercus semecarpifolia* Sm, *Pyrus pashia* Buch. & Ham and *Schima wallichii* Choisy were selected for the present investigation. These were collected from the community forest of Chitang, Makwanpur district situated at 27° 36′ 29″ N, 85° 5′ 39″ E at 1500–2500 m a.s.l., in the central hills of Nepal. The field visit was done in July, 2018 and sample collection was done as per the guideline of the document received from the Central Department of Botany, Tribhuvan University, Nepal. The plant was identified by Dr. Keshav Raj Rajbhandri. A voucher specimen of this plant is deposited in the Tribhuvan University Central Herbarium (TUCH) (voucher number – M05). Roots and leaves were excised from the selected three individual plants without harming the plants and wrapped with tissue paper in the well labeled zip bags and brought to laboratory and further steps carried out immediately.

### Isolation of endophytic fungi

Collected roots and leaves from different individuals of *D. longicornu* were surface sterilized [6]. The outer epidermal layer was removed to avoid contamination and cut into 2 cm long sections with a sterile scalpel. Explants were excised using sterile scalpel blade further rinsed 3-4 times with sterile distilled water. Excess of water from explants was removed using sterile tissue paper. A portion of the sterile distilled water from the last wash was poured in the PDA Petri dish to check for the efficiency of the sterilization procedure.

Czapek Dox agar (CDA), Potato Dextrose agar (PDA) and Modified Melin-Norkrans (MMN) media were used for isolation of endophytic fungi. Each experiment was performed in triplicate for every media, each plate contained 4 explants. An explant was placed in the media and gently tapped once to ensure that cut portion should touch medium surface. Inoculated media were incubated at 25 °C for 7 days in dark. The fungal cultures were observed selected based on the growth pattern, color, and texture of the colony. The cultures were obtained in pure culture form and preserved in laboratory for further use. The pure cultures were stored in a Petri plate as well as in 20% glycerol stock in a vial at 4 °C.

### Molecular identification of endophytic fungi

Molecular identification of each endophyte purely isolated in the petridish was considered. Genomic DNA has been extracted from 50 mg mycelia by Cetyltrimethylammonium bromide (CTAB) method [6, 51]. Extracted genomic DNA was visualized using 1% gel electrophoresis. For PCR amplification of ITS region, the primer set ITS1 (5′-TCC GTA GGT GAA CCT GCG G-3′) and ITS4 (5′ TCC TCC GCT TAT TGA TAT GC 3′) was used. The PCR conditions were programmed as follows: one cycle of 4 min at 95 °C, followed by 35 cycles at 95 °C for 30 s, 53 °C for 30 s, 72 °C for 1 min 30 s, and extension at 72 °C for 7 min. Negative controls (absence of DNA template) were used to detect DNA contamination [6, 52, 53]. The ITS gene sequencing was carried out using on ABI 3730 XL DNA analyzer (Applied Biosystems Inc., USA) using the ABI Big-Dye terminator version 3.1 sequencing kit as per the manufacturer’s instructions. The consensus ITS sequence of each identified fungus were generated by using Chromas Pro 2.1.8 (Technelysium Pty Ltd., Australia) and deposited to NCBI GenBank database. Further, these ITS sequences were compared with available ITS sequences of the fungi in the NCBI database using NCBI BLAST tool online (https://blast.ncbi.nlm.nih.gov/Blast.cgi).

### Amplification of iaaM gene

Amplification of *iaaM* gene was done with a pair of primer forward 5′-AGTGACCAGCCTGCTGATTTCCCTCG-3′and reverse 5′-AAGATCGCAGCCATTGAGTTGTGC-3′. The PCR amplifications were performed using Veriti thermal cycler (Applied Bio-system, Singapore). Optimized PCR condition for amplification of *iaaM* gene were as follows: initial denaturation at 94 °C for 5 min, denaturation at 94 °C for 1 min, annealing at 57 °C for 1 min, extension at 72 °C for 1 min and a final extension at 72 °C for 10 min. Thirty-five cycles were carried out for each genomic DNA [22, 54].

### Quantifications of IAA in fungal extract

The IAA quantification for each fungal culture extract was done as described previously [6]. In this regard, the fungi were incubated in 20 mL of Cazepek Dox broth with or without supplementation of 1 mg of DL-tryptophan. After incubation, Salkowski reagent was added to the filtrate of broth (1:2 ratio) followed by incubation in dark for 25 min and the absorbance was measured at 530 nm in an UV-VIS spectrophotometer. Recorded absorbance was compared with the standard IAA curve to find the concentration of IAA in extract. Three independent biological replicates were taken for the quantification [6].

### Quantification of the IAA by HPLC

Fungal isolates were cultured separately for 20 days in 250 mL Potato Dextrose broth supplemented with 2 mg DL-trytophan (Mumbai, Himedia). The broths were washed three times with equal volume of ethyl acetate. The organic extract was separated out and then dried followed by suspension in HPLC graded methanol (Fisher Scientific, Mumbai, India) and filtered by syringe filter (0.22 μm).

The instrument that was used for the quantification was High performance liquid chromatography (HPLC) (Shimadza UFLC DGU-20 A5R) Japan and the column was C18 shim-pack column size (5μmX 4.6X150mm); analysis parameter were oven temperature at 30 °C throughout the experiment; absorbance of Indole acetic acid 280 nm; Solvent phase 0 to 55% acetonitrile for 25 min. Then 100% acetonitrile for 28 min. 0.1% acetic acid in water flow rate 1.5 ml per min [55]. The retention time for the IAA present the samples were compared with the standard graph of IAA.

### Biochemical assay

Plantlet extract were prepared for the Biochemical assay. In this regard, 60 days old in vitro grown plantlets of *C. aloifolium,* uncolonized as well as colonized by DLCCR3, DLMR3 and DLCCR7 were taken separately grinded, with the help of mortar and pestle [23]. The extract was suspended in HPLC grade methanol (Fisher Scientific) for a day to extract bioactive compounds. The methanol extract was then further filtered by Whatman filter paper Grade 1:11 μm and taken for chemical profiling by GC-MS technique.

Similarly, the DLCCR3, DLMR3 and DLCCR7 fungal extract were prepared as described previously by Shah et al. [6]. After 15 days of incubation in Potato dextrose broth, fungal broth of DLCCR3, DLMR3 and DLCCR7 were processed for the extraction. The broth was centrifuged at 10,000 rpm for 30 min followed by filtration with whatman filter paper Grade 1:11 μm and pH adjusted to 2.5 with 1 N HCl. The filtrates were then treated with equal volume of HPLC-grade ethyl acetate (Fisher Scientific, Mumbai, India), shaken. The process was repeated three times and using a separating funnel collected the organic extracts. The extracts were then subjected to a rotary evaporator, (DLAB: RE100-Pro) and dried at 40 °C. The residue was then re-suspended in 2 mL of HPLC-grade methanol (Fisher Scientific, Mumbai, India) for bioactive compound detection.

The bioactive compounds present in the samples of uncolonised plant extract; colonized plant extract with DLCCR3; DLMR3; DLCCR7 as well as fungal extract DLCCR3; DLMR3 and DLCCR7 were identified separately by GCMS technique, GC-MS-QP2010 Ultra (Shimadzu Europa GmbH, Germany) instrument fitted with RTX-5MS (30 × 0.25 × 0.10 m) column. The parameters were set up as: The instrument initial final temperatures 100 °C and 250 °C, respectively. Rate of Helium flow was 1 mL min^− 1^ at 0.80 KV of ionization voltage. The sample injection was done split less mode. Mass spectral scan ranged from30 to 600 (m/z). The obtain chromatogram was then compared with the library of National Institute of Standard and Technology, NIST, US [23, 25].

### Plant growth assay with fungal elicitor

For the plant growth assay the fungal elicitor was prepared as described previously [6]. The MS media supplemented with fungal elicitor was prepared (0.4 mL fungal extract in 19.6 mL MS media). The in vitro grown protocorms of *D. longicornu* grown from aseptic seed in basal MS media were used for the plant growth assay and the MS basal medium as control. The assay was monitor for 45 days under a 16 h photoperiod at 25 °C ± 2 °C. The growth pattern such as number of shoots, roots and their length were recorded.

### Plant growth assay with fungus

For the co-culture assay, plantlets *C. aloifolium* were grown in-vitro aseptic seeds in basal MS medium in the laboratory. The in vitro culture of *C. aloifolium* was followed as described previously [12]. The fully-grown plantlets were allowed to grow for 60 days along with 5 mg mycelium of the fungi DLCCR3, DLMR3 and DLCCR7 separate culture bottles. The plantlet in basal medium without fungal inoculation was taken as control. The assay was monitor under a 16 h photoperiod at 25 °C ± 2 °C. The plant growth was monitored and the number of shoots, roots and their length were recorded and compared with that of control.

### Histo-chemical microscopy

The root sections of the plantlets interacted with the fungi was subjected to histo-chemical microscopy. The protocol trypan blue root section staining was done as described [25]. The stained root sections were observed under bright field microscope. The interacted root section was also examined by analytical scanning electron microscopy (JEOL JSM-6360A). The root section was subjected dehydration with series of ethanol concentration as described [54].

### Acclimatization

The co-cultured plantlets were then acclimatized with coco peat and moss in ratio of 1:2 [23, 51]. The plantlets were frequently moistened and monitored for their growth and development. The growth pattern of the plantlet was observed for 60 days and compared with that of control.

### Statistical analysis

Quantification of IAA from the fungal extract, IAA standard curve were prepared by using Microsoft excel 2007 and SPSS statistic 20. The quantification of IAA from fungal extract was done independently for three replicates. Plant growth assays were performed independently for five replicates, number of plantlets were 15. The plant growth patterns in terms of shoot and root number as well as their length were calculated. The bar diagrams with standard mean and standard error were taken (mean ± SE; *n* = 15). One-way analysis of variance was done at the significant level of *p* ≤ 0.05 (Tukey HSD test).

## Supplementary Information

**Additional file 1: Figure S1.** The amplification of 400 bp *iaa*M gene from the fungal specimen DLCCR7, DLCCR3 and DLMR3. **Figure S2.** The Chromatogram of HPLC showing the peak of Standard IAA (a); Sample DLCCR7 (b), DLCCR3 (c) and DLMR3 (d). **Figure S3.** The GCMS chromatogram of the methanol extract of uncolonized plant. **Table S1.** List of the compounds identified from methanol extract of uncolonized plant. **Figure S4.** Chromatogram of the bioactive compounds present in Plant–DLMR3. **Table S2.** List of the bioactive compounds present in Plant- DLMR3. **Figure S5.** The chromatogram of the Plant-DLCCR7. **Table S3.** List of the bioactive compounds in Plant–DLCCR7. **Figure S6.** Chromatogram of bioactive compounds of Plant-DLCCR3. **Table S4.** List of the bioactive compounds present in Plant-DLCCR3. **Figure S7.** Chromatogram of the bioactive compounds of DLCCR3. **Table S5.** List of Bioactive compounds present in DLCCR3. **Figure S8.** Chromatogram of Bioactive compounds present in DLMR3. **Table S6.** List of bioactive compounds present in DLMR3 extract. **Figure S9.** Chromatogram of bioactive compounds present in DLCCR7. **Table S7.** List of bioactive compounds present in DLCCR7.

## Data Availability

All data generated and analyzed are available in this published article and the Supplementary data file 1.

## Declarations

The field visit was done in July, 2018 and sample collection was done as per the guideline of the document received from the Central Department of Botany, Tribhuvan University, Nepal. The plant was identified by Dr. Keshav Raj Rajbhandri. A voucher specimen of this plant is deposited in the Tribhuvan University Central Herbarium (TUCH) (voucher number – M05).
