# Supplementary material for: Colonization with non-mycorrhizal culturable endophytic fungi enhances orchid growth and indole acetic acid production
Source: BMC Microbiol. 2022 Apr 13;22:101. doi: 10.1186/s12866-022-02507-z (PMC9006483; doi:10.1186/s12866-022-02507-z)
Supplement: Supplementary file 1 — Additional file 1: Figure S1. The amplification of 400 bp iaaM gene from the fungal specimen DLCCR7, DLCCR3 and DLMR3. Figure S2. The Chromatogram of HPLC showing the peak of Standard IAA (a); Sample DLCCR7 (b), DLCCR3 (c) and DLMR3 (d). Figure S3. The GCMS chromatogram of the methanol extract of uncolonized plant. Table S1. List of the compounds identified from methanol extract of uncolonized plant. Figure S4. Chromatogram of the bioactive compounds present in Plant–DLMR3. Table S2. List of the bioactive compounds present in Plant- DLMR3. Figure S5. The chromatogram of the Plant-DLCCR7. Table S3. List of the bioactive compounds in Plant–DLCCR7. Figure S6. Chromatogram of bioactive compounds of Plant-DLCCR3. Table S4. List of the bioactive compounds present in Plant-DLCCR3. Figure S7. Chromatogram of the bioactive compounds of DLCCR3. Table S5. List of Bioactive compounds present in DLCCR3. Figure S8. Chromatogram of Bioactive compounds present in DLMR3. Table S6. List of bioactive compounds present in DLMR3 extract. Figure S9. Chromatogram of bioactive compounds present in DLCCR7. Table S7. List of bioactive compounds present in DLCCR7. [file 12866_2022_2507_MOESM1_ESM.docx]

**
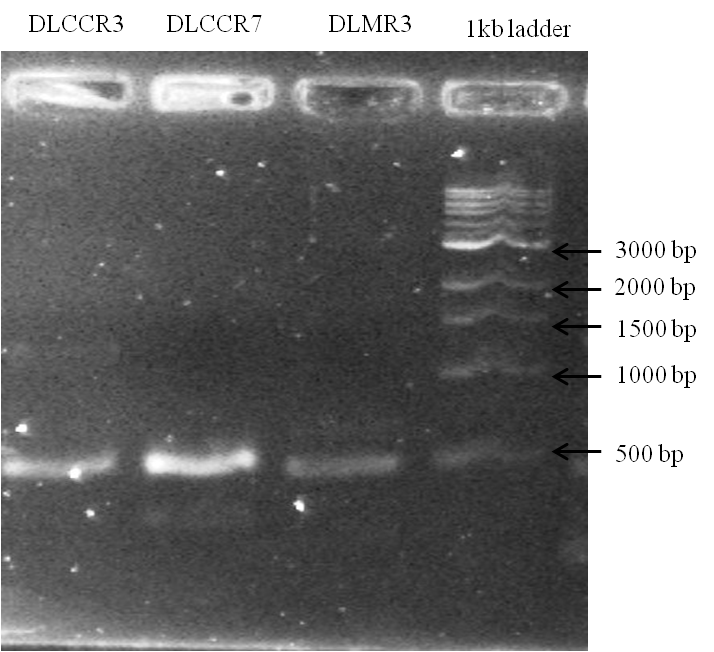
**

**Figure S1.** The amplification of 400bp *iaa*M gene from the fungal specimen DLCCR7, DLCCR3 and DLMR3

**
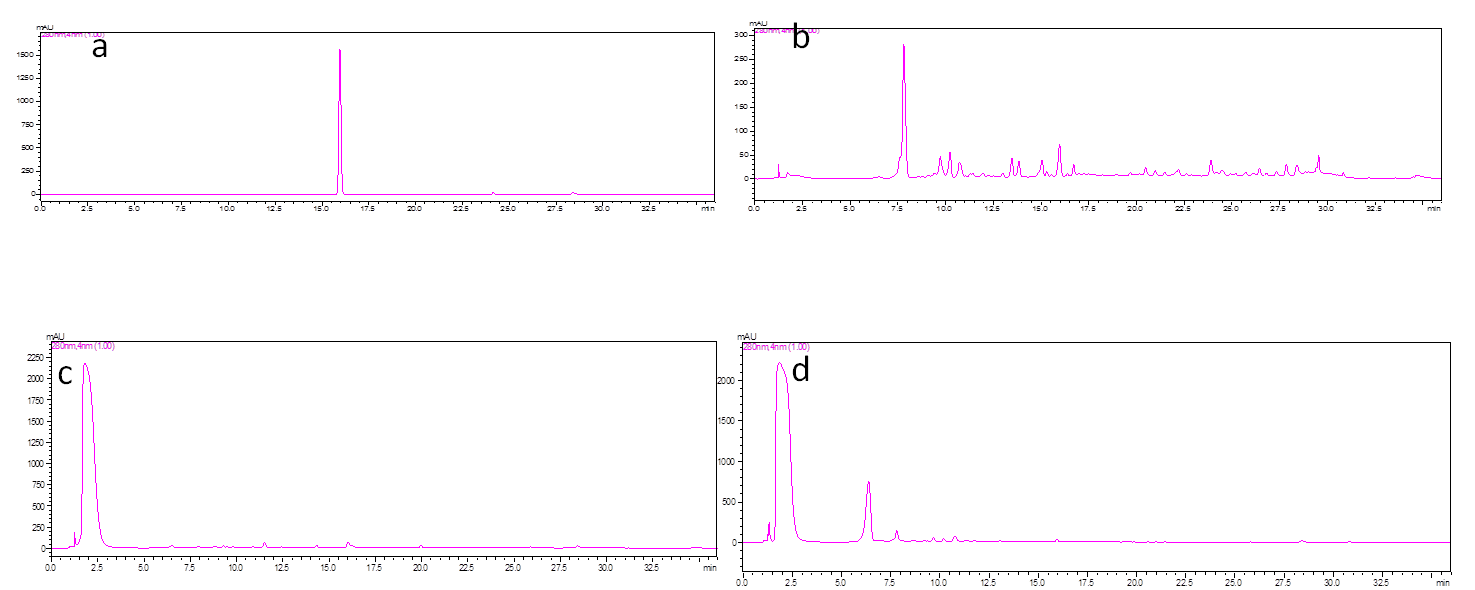
 Figure S2.** The Chromatogram of HPLC showing the peak of Standard IAA (**a**); Sample DLCCR7 (**b**), DLCCR3 (**c**) and DLMR3 (**d**).

**Figure S3.** The GCMS chromatogram of the methanol extract of uncolonized plant.

**Table S1.** List of the compounds identified from methanol extract of uncolonized plant.

| **Peak** | **Retention Time** | **Name** | **Mass peak** | **Base Peak** |
| --- | --- | --- | --- | --- |
| 1. | 10.410 | Hexadecanoic acid, methyl ester | 640 | 74.10 |
| 2. | 11.125 | Heptadecanoic acid, heptadecyl ester | 586 | 43.15 |
| 3. | 12.930 | Hexadecanoic acid, 1-(hydroxymethyl)-1,2-ethanediyl ester | 588 | 57.15 |
| 4. | 13.580 | d-Mannitol, 1-O-(22-hydroxydocosyl)- | 613 | 73.10 |
| 6. | 17.850 | Ethyl iso-allocholate | 685 | 55.10 |
| 7. | 19.445 | d-Mannitol, 1-O-(22-hydroxydocosyl)- | 704 | 73.10 |
| 8. | 25.975 | beta. Carotene | 820 | 55.10 |
| 9. | 28.080 | Ethyl iso-allocholate | 685 | 43.15 |

**\**

**Figure S4.** Chromatogram of the bioactive compounds present in Plant–DLMR3.

**Table S2:** List of the bioactive compounds present in Plant- DLMR3.

| Peaks | R. Time | Area% | Name | Base m/z |
| --- | --- | --- | --- | --- |
| 1 | 8.311 | 0.29 | Z- 1,9-Hexadecadiene | 41.05 |
| 2 | 8.833 | 0.78 | Cyclopropane | 41.05 |
| 3 | 9.178 | 1.64 | Pentadecanoic acid | 74.10 |
| 4. | 9.567 | 6.67 | n-Hexadecanoic acid | 41.05 |
| 5 | 9.773 | 1.94 | Cyclopropane | 63.05 |
| 6 | 9.842 | 1.73 | Cyclopentaneundecanoic acid | 55.05 |
| 7 | 10.325 | 2.30 | 11, 14-Eicosadienoic acid | 41.10 |
| 8 | 11.387 | 6.45 | 1,2,4-Triazole | 75.05 |
| 9 | 12.660 | 0.48 | d-Mannitol | 69.05 |

**Figure S5.** The chromatogram of the Plant-DLCCR7.

**Table S3.** List of the bioactive compounds in Plant–DLCCR7

| Peak | Real Time | Area% | Name | Base m/z |
| --- | --- | --- | --- | --- |
| 1 | 3.513 | 0.83 | Imidazole | 43.95 |
| 2 | 8.490 | 0.70 | Hexadecanoic acid | 74.10 |
| 3 | 8.546 | 0.51 | Cyclopropane | 41.10 |
| 4 | 9.073 | 0.45 | Cyclopropanepentanoic acid | 41.10 |
| 5 | 9.188 | 7.34 | Pentadecanoic acid | 74.10 |
| 6 | 10.027 | 0.56 | 2H-Pyran | 63.05 |
| 7 | 9.564 | 7.00 | n-Hexadecanoic acid | 41.10 |
| 8 | 10.772 | 11.06 | Oleic acid | 41.05 |

**Figure S6.** Chromatogram of bioactive compounds of Plant-DLCCR3.

**Table S4.** List of the bioactive compounds present in Plant-DLCCR3.

| Peak | Real Time | Area % | Name | Base m/z |
| --- | --- | --- | --- | --- |
| 1 | 9.175 | 2.31 | Pentadecanoic acid | 74.10 |
| 2 | 9.913 | 1.08 | Cyclopropane | 63.00 |
| 3 | 10.563 | 6.07 | 2H-Pyran-5-carboxamide | 63.05 |

**Figure S7.** chromatogram of the bioactive compounds of DLCCR3.

**Table S5.** List of Bioactive compounds present in DLCCR3.

| Peak | Real Time | Area% | Name | Base m/z |
| --- | --- | --- | --- | --- |
| 1 | 9.553 | 7.21 | n-Hexadecanoic acid | 41.05 |
| 2 | 10.626 | 2.20 | Cyclopropane | 41.05 |
| 3 | 10.759 | 16.10 | Oleic Acid | 41.05 |

**Figure S8.** Chromatogram of Bioactive compounds present in DLMR3.

**Table S6.** List of bioactive compounds present in DLMR3 extract.

| Peak | Real Time | Area % | Name | Base m/z |
| --- | --- | --- | --- | --- |
| 1 | 4.169 | 44.41 | 4-Hepten-3-one, 4-methyl- | 41.05 |
| 2 | 6.217 | 6.27 | 2H-Pyran-2-one | 42.05 |
| 3 | 7.790 | 4.15 | 5-Methyl-2-methylamino-2-thiazoline | 42.00 |
| 4 | 10.740 | 19.60 | Cyclopentaneundecanoic acid | 41.05 |
| 5 | 14.840 | 2.51 | 6-Acetyl-.beta.-d-mannose | 55.10 |

**Figure S9.** Chromatogram of bioactive compounds present in DLCCR7.

**Table S7.** List of bioactive compounds present in DLCCR7.

| Peak | Real Time | Area % | Name | Base m/z |
| --- | --- | --- | --- | --- |
| 1 | 4.392 | 15.77 | 2-Furancarboxaldehyde, 5-(hydroxymethyl)- | 41.05 |
| 2 | 6.269 | 46.30 | 4H-Pyran-4-one | 142.20 |
| 3 | 9.545 | 4.24 | n-Hexadecanoic acid | 43.10 |
| 4 | 10.495 | 3.31 | Cyclopentaneundecanoic acid | 41.10 |
| 5 | 10.123 | 1.25 | Cyclopropanecarboxylic acid | 53.20 |
| 6 | 10.744 | 12.00 | Oleic Acid | 41.10 |
| 7 | 11.424 | 1.61 | 2,3,4,6-Tetra-O-acetyl-D-glucopyranose | 75.10 |
